# Association of SPISE with prevalent and incident MASLD: a two-stage population-based study and development of a risk prediction model

**DOI:** 10.3389/fnut.2026.1811730

**Published:** 2026-04-29

**Authors:** Yang Cheng, Jingzhi Wang

**Affiliations:** 1 Department of Hepatobiliary Surgery, Clinical Oncology School of Fujian Medical University, Fujian Cancer Hospital, Fuzhou, Fujian, China; 2 Department of Radiotherapy Oncology, The First People's Hospital of Yancheng, Yancheng No.1 People's Hospital, Affiliated Hospital of Medical School, Nanjing University, Yancheng, China

**Keywords:** cohort study, decision curve analysis, MASLD, nomogram, risk prediction, SPISE, time-dependent ROC

## Abstract

**Background:**

The prevalence of metabolic dysfunction–associated steatotic liver disease (MASLD) is increasing, and the disease is often asymptomatic in its early stages. Insulin resistance is central, but fasting-insulin–based indices are impractical for routine screening. The single-point insulin sensitivity estimator (SPISE) is simple and inexpensive, but its longitudinal association with MASLD and predictive utility remain unclear.

**Methods:**

A two-stage study was performed. Cross-sectional analyses examined SPISE and prevalent MASLD; longitudinal analyses among participants without MASLD at baseline assessed incident MASLD and developed prediction models. MASLD was defined as ultrasound-confirmed steatosis plus ≥1 cardiometabolic risk factor. Logistic regression estimated odds ratios (ORs) for prevalent MASLD; Hazard ratios (HRs) for incident MASLD were estimated using Cox proportional hazards models, with restricted cubic splines used to characterize non-linear exposure–response patterns. Improvement in prediction after adding SPISE was compared with TyG, METS-IR, and TG/HDL-C using NRI and IDI metrics. Discriminative ability and clinical net benefit were examined with time-dependent ROC analysis and decision curve analysis, respectively.

**Results:**

Higher SPISE was independently associated with lower odds of prevalent MASLD (fully adjusted OR = 0.43, 95% CI 0.38–0.48). Higher SPISE also predicted lower incident MASLD risk (fully adjusted HR = 0.49, 95% CI 0.46–0.52), with a significant nonlinear association (P for non-linearity < 0.001). Adding SPISE to the fully adjusted base model produced the largest improvements in reclassification and discrimination (NRI = 0.363; IDI = 0.093; both *p* < 0.001). A SPISE-based model incorporating liver enzymes, bilirubin, and blood pressure showed good discrimination at 12 and 24 months (AUC = 0.859 and 0.886, respectively) and favorable clinical net benefit.

**Conclusion:**

SPISE is independently and inversely associated with prevalent and incident MASLD and provides superior incremental predictive value versus common insulin resistance indices. External validation is warranted.

## 1 Introduction

Metabolic dysfunction–associated steatotic liver disease (MASLD) represents a leading cause of chronic liver disease globally, with prevalence rising alongside increasing rates of obesity and cardiometabolic disorders worldwide (1–3). MASLD may advance to fibrosis, cirrhosis, and hepatocellular carcinoma and is associated with greater risks of extrahepatic complications such as cardiovascular and chronic kidney disease, contributing to an increasing public health burden (4–8). Given that MASLD is frequently asymptomatic at early stages and affects a large population, scalable tools for risk identification and stratification are urgently needed to facilitate early intervention and optimize resource allocation.

Although liver biopsy remains an important reference standard, its invasiveness, high cost, and susceptibility to sampling variability limit its suitability for population-level screening and long-term follow-up (9, 10). Noninvasive imaging modalities such as elastography enable assessment of steatosis and fibrosis; however, limited accessibility and operator dependence restrict broad implementation in community settings and resource-constrained environments (9). Consequently, low-cost and scalable risk assessment strategies based on routinely available clinical and laboratory measures remain a key unmet need for advancing earlier prevention and control of MASLD.

Insulin resistance is widely regarded as a central pathophysiological mechanism underlying the development and progression of MASLD (11). Conventional insulin resistance indices often require fasting insulin measurement, which hampers routine use in clinical practice and health examinations (12). The single-point insulin sensitivity estimator (SPISE) is a pragmatic proxy for insulin sensitivity, derived from routinely collected body mass index (BMI), triglycerides (TG), and high-density lipoprotein cholesterol (HDL-C) (13). Previous studies have linked SPISE to adverse cardiometabolic outcomes (14, 15); nevertheless, longitudinal evidence for MASLD—particularly incident MASLD—and its added predictive value remains limited.

Most existing studies evaluating metabolic indices in relation to MASLD have been cross-sectional (16–18), leaving concerns regarding reverse causation and residual confounding. Whether SPISE independently predicts future MASLD risk and provides incremental predictive utility when incorporated into multivariable models warrants further clarification. To fill these evidence gaps, a two-stage discovery–validation design was implemented, first examining SPISE in relation to prevalent MASLD in cross-sectional analyses and then confirming the baseline SPISE–incident MASLD association prospectively in an independent cohort. In addition, a prediction model for incident MASLD was derived and validated by integrating SPISE with key clinical measures, with the goal of delivering an inexpensive and readily deployable approach to support early detection and stratified management across healthcare and community settings.

## 2 Materials and methods

### 2.1 Data sources and ethical approval

This work reanalyzed public, fully de-identified data. The cross-sectional component used a Dryad-hosted dataset from a single-center retrospective study by Yan et al. (DOI: https://doi.org/10.5061/dryad.7d7wm3809) (19). That parent study received approval from the Institutional Review Board of Tongji Medical College, Huazhong University of Science and Technology (IRB: S155) (19), and reported that informed consent had been obtained. The longitudinal component relied on an open-access follow-up cohort published by Sun et al. (20); the source study was approved by the Ethics Committee of Wenzhou People’s Hospital and likewise documented informed consent. Because the present analyses involved only anonymized records and required no new enrollment or data acquisition, re-consent was not required. Data use conditions were observed, and the study adhered to the Declaration of Helsinki.

### 2.2 Study population and design

The study proceeded in two phases. Phase 1 assessed the association of SPISE with baseline MASLD in a cross-sectional sample. Phase 2 used follow-up data to verify the SPISE–incident MASLD relationship and to construct risk prediction models.

For the cross-sectional stage, eligible participants were identified from the Dryad dataset of Yan F et al. Participants with missing key variables required for SPISE calculation or outcome definition were excluded, yielding 1,592 individuals for analysis (19).

For longitudinal validation, the follow-up cohort reported by Sun DQ et al. was used. From a health-check population, 16,173 non-obese individuals free of MASLD at enrollment were included and followed longitudinally (20). Participants were excluded at baseline for any of the following (20): missing/incomplete clinical data with unsuccessful follow-up; current use of oral antihypertensive, lipid-lowering, or glucose-lowering agents; heavy alcohol intake (men ≥140 g/day; women ≥70 g/day); pre-existing MASLD, autoimmune hepatitis, viral hepatitis, or other chronic liver disease of known etiology; LDL-C > 3.12 mmol/L; or BMI ≥ 25 kg/m^2^. Based on the public dataset, after calculation of the exposure index and harmonization of covariates, participants with missing key variables were additionally excluded, resulting in 16,172 individuals included in longitudinal analyses. The endpoint was the first diagnosis of MASLD during follow-up.

### 2.3 Definitions and measurements

The primary exposure was the SPISE, calculated using a prespecified formula based on BMI, TG, and HDL-C. SPISE was analyzed as a continuous variable and in tertiles (T1–T3). For comparative assessment, the METS-IR (21), TG/HDL-C ratio (22), and the TyG index (22) were computed according to published formulas with harmonized units.

The outcome was MASLD. Hepatic steatosis in both datasets was assessed by abdominal ultrasonography and defined by the presence of ≥2 of the following (23): diffusely increased hepatic echogenicity, increased hepatorenal contrast, and posterior beam attenuation with poor visualization of deep intrahepatic vessels. MASLD was operationalized as ultrasound-confirmed steatosis plus ≥1 cardiometabolic risk factor assessed at baseline. Cardiometabolic risk was considered present if at least one of the following criteria was met (24): BMI ≥ 23.0 kg/m^2^; abnormal glycemia/diabetes (fasting plasma glucose ≥5.6 mmol/L or self-reported diabetes); dyslipidemia (TG ≥ 1.7 mmol/L or reduced HDL-C: men <1.0 mmol/L, women <1.3 mmol/L); or elevated blood pressure (SBP ≥ 130 mmHg or DBP ≥ 85 mmHg). Prevalent MASLD was defined at baseline in cross-sectional analyses; incident MASLD was defined as first meeting the criteria during follow-up among those free of MASLD at baseline.

Prespecified covariates included age, sex, blood pressure, liver-related biomarkers (ALT, AST, GGT, ALP, total bilirubin [TB], direct bilirubin [Dbil], albumin [ALB], globulin [GLB]), kidney-related biomarkers (creatinine [Cr], blood urea nitrogen [BUN], uric acid [UA]), and other routinely measured laboratory variables available in the datasets (25–27).

### 2.4 Statistical analysis

All statistical procedures were performed in R (version 4.5.1), applying two-tailed tests with a significance threshold of *p* < 0.05. Depending on distribution assessed by the Shapiro–Wilk test, continuous variables were reported as mean ± SD or median (IQR) and compared using Student’s *t* test or the Mann–Whitney *U* test. Categorical variables were summarized as *n* (%) and evaluated using the *χ*^2^ test or Fisher’s exact test.

For the cross-sectional analysis, adjusted logistic regression models were fitted to quantify associations between SPISE and prevalent MASLD, reporting ORs with 95% CIs for SPISE modeled continuously and as tertiles. Dose–response trends were evaluated by entering SPISE tertiles as an ordered variable. For longitudinal analyses, Kaplan–Meier estimates were generated by tertile and compared using log-rank tests. Associations with incident MASLD were then quantified using Cox proportional hazards regression, reporting HRs with 95% CIs. A prespecified sequential adjustment strategy was applied (unadjusted; age- and sex-adjusted; fully adjusted for available clinical and laboratory covariates). The proportional hazards assumption was examined using Schoenfeld residuals. Nonlinear exposure–response patterns were explored by fitting restricted cubic spline terms in the fully adjusted Cox model, with statistical tests reported for both the global association and the nonlinear component.

Incremental predictive value was examined by entering SPISE, TyG, METS-IR, and TG/HDL-C one at a time into the fully adjusted reference model; changes in prediction were quantified using NRI and IDI with 95% CIs.

Model development used the longitudinal cohort. Candidate predictors were prespecified from the fully adjusted model, and variable selection was performed using LASSO with cross-validated *λ* (28). Variables retained after selection were refitted in a multivariable Cox model to generate the final risk equation, which was then translated into a nomogram and a web-based dynamic tool for estimating 12- and 24-month incident MASLD risk. Discrimination was quantified using time-dependent ROC curves and AUC. At 24 months, decision curve analysis was used to examine net benefit across a range of risk thresholds, benchmarking the model against the “treat-all” and “treat-none” approaches.

As a sensitivity analysis, participants who developed MASLD within the first 12 months of follow-up were excluded, and the Cox regression and restricted cubic spline analyses were repeated to reduce potential reverse causation and possible misclassification related to occult baseline steatosis. In addition, to further assess the robustness and internal validity of the final prediction model, internal cross-validation was performed for the 12- and 24-month models, and model performance was evaluated using the AUC and the Brier score.

## 3 Results

### 3.1 Baseline characteristics of the cross-sectional population

MASLD was identified in 744 of 1,592 participants (46.7%). Compared with non-MASLD individuals (*n* = 848), the MASLD subgroup showed male predominance and a uniformly less favorable cardiometabolic profile, including higher BMI and blood pressure (SBP/DBP), higher fasting plasma glucose and TG, and lower HDL-C (all *p* < 0.001). Hepatic transaminase activities (ALT and AST) were also increased (all *p* < 0.001). Consistently, SPISE was lower in the MASLD group (5.31 ± 1.13 vs. 6.89 ± 1.42, *p* < 0.001) (Table 1).

**Table 1:** Characteristics of the study population in the cross-sectional analysis.

Variable	Overall (*N* = 1,592)	Non-MASLD (*N* = 848)	MASLD (*N* = 744)	*p* value
Male sex, *n* (%)	1,148 (72.11)	546 (64.39)	602 (80.91)	**<0.001**
Age group, *n* (%)				0.083
40–49	354 (22.24)	176 (20.75)	178 (23.92)	
50–59	709 (44.54)	373 (43.99)	336 (45.16)	
60–69	360 (22.61)	195 (23.00)	165 (22.18)	
70–79	169 (10.62)	104 (12.26)	65 (8.74)	
T2DM, *n* (%)	498 (31.28)	163 (19.22)	335 (45.03)	**<0.001**
Hypertension, *n* (%)	943 (59.23)	421 (49.65)	522 (70.16)	**<0.001**
Alcohol use, *n* (%)	538 (33.79)	240 (28.30)	298 (40.05)	**<0.001**
Tobacco use, *n* (%)	520 (32.66)	228 (26.89)	292 (39.25)	**<0.001**
BMI (kg/m^2^)	25.41 ± 2.92	24.41 ± 2.59	26.54 ± 2.86	**<0.001**
SBP (mmHg)	130.94 ± 15.82	127.50 ± 15.13	134.86 ± 15.68	**<0.001**
DBP (mmHg)	81.60 ± 11.19	78.79 ± 10.50	84.80 ± 11.10	**<0.001**
ALT (U/L)	21.00 (15.00, 30.00)	18.00 (13.00, 25.00)	26.00 (19.00, 36.25)	**<0.001**
AST (U/L)	23.13 ± 10.97	21.49 ± 9.41	25.00 ± 12.25	**<0.001**
UA (μmol/L)	366.75 ± 95.83	344.89 ± 88.09	391.67 ± 98.23	**<0.001**
FBG (mg/dL)	5.54 ± 1.65	5.10 ± 1.27	6.05 ± 1.88	**<0.001**
TC (mmol/L)	4.48 ± 1.09	4.35 ± 1.00	4.63 ± 1.17	**<0.001**
TG (mmol/L)	1.42 (0.98, 2.18)	1.12 (0.84, 1.47)	2.05 (1.42, 2.90)	**<0.001**
HDL-C (mmol/L)	1.11 ± 0.32	1.20 ± 0.34	1.02 ± 0.26	**<0.001**
LDL-C (mmol/L)	2.67 ± 0.89	2.64 ± 0.86	2.70 ± 0.92	0.153
SPISE	6.15 ± 1.52	6.89 ± 1.42	5.31 ± 1.13	**<0.001**

Values are presented as *n* (%), mean ± SD, or median (IQR), as appropriate. BMI, body mass index; T2DM, type 2 diabetes mellitus; DBP, diastolic blood pressure; SBP, systolic blood pressure; AST, aspartate aminotransferase; TG, triglyceride; ALT, alanine aminotransferase; LDL-C, low-density lipid cholesterol; HDL-C, high-density lipoprotein cholesterol; TC, total cholesterol; FBG, fasting blood glucose; UA, uric acid; MASLD, metabolic dysfunction-associated steatotic liver disease; SPISE, single-point insulin sensitivity estimator. *p*-value less than 0.05 is expressed in bold.

### 3.2 Cross-sectional association between SPISE and prevalent MASLD

In the unadjusted model, SPISE was inversely associated with prevalent MASLD (continuous SPISE: OR = 0.37, 95% CI 0.33–0.41). This association remained unchanged after adjustment for age and sex (OR = 0.37, 95% CI 0.33–0.41). In the fully adjusted model, SPISE remained independently associated with lower odds of MASLD (OR = 0.43, 95% CI 0.38–0.48) (Table 2).

**Table 2:** Cross-sectional association of SPISE with MASLD.

Variable	Model 1		Model 2		Model 3	
	OR (95% CI)	*p* value	OR (95% CI)	*p* value	OR (95% CI)	*p* value
SPISE continuous	0.37 (0.33–0.41)	<0.001	0.37 (0.33–0.41)	<0.001	0.43 (0.38–0.48)	<0.001
SPISE tertiles						
T1	1.00 [Ref]		1.00 [Ref]		1.00 [Ref]	
T2	0.22 (0.17–0.29)	<0.001	0.22 (0.17–0.29)	<0.001	0.30 (0.22–0.40)	<0.001
T3	0.06 (0.05–0.09)	<0.001	0.07 (0.05–0.09)	<0.001	0.12 (0.08–0.17)	<0.001
*p* for trend	0.25 (0.22–0.29)	<0.001	0.26 (0.22–0.31)	<0.001	0.34 (0.29–0.40)	<0.001

Model 1: no covariates were adjusted. Model 2: gender, age were adjusted. Model 3: gender, age, ALT, AST, UA, SBP, DBP, tabacco use, alcohol use, hypertension, diabetes. BMI, body mass index; AST, aspartate aminotransferase; UA, uric acid; TB, total bilirubin; SBP, systolic blood pressure; DBP, diastolic blood pressure; MASLD, metabolic dysfunction-associated steatotic liver disease; SPISE, single-point insulin sensitivity estimator.

When analyzed by SPISE tertiles, and using the lowest tertile (T1) as the reference, the fully adjusted ORs were 0.30 (95% CI 0.22–0.40) for T2 and 0.12 (95% CI 0.08–0.17) for T3 (*p* for trend < 0.001), indicating a clear dose–response gradient between higher SPISE and lower odds of prevalent MASLD (Table 2).

### 3.3 Baseline characteristics and follow-up outcomes in the longitudinal cohort

The longitudinal cohort included 16,172 participants free of MASLD at baseline, among whom 2,000 incident MASLD cases occurred during follow-up. Compared with participants who did not develop MASLD, those with incident MASLD were older and more often male. Baseline BMI, SBP, and DBP were also higher in the incident MASLD group (all *p* < 0.001) (Table 3).

**Table 3:** Baseline characteristics of the study population in the longitudinal analysis.

Variable	Overall (*N* = 16,172)	Non-MASLD (*N* = 14,172)	MASLD (*N* = 2,000)	*p* value
Age (year)	43.23 ± 14.96	43.00 ± 14.91	44.83 ± 15.23	**<0.001**
Male sex, *n* (%)	8,483 (52.45)	7,345 (51.83)	1,138 (56.9)	**<0.001**
BMI (kg/m^2^)	21.38 ± 2.05	21.09 ± 1.98	23.41 ± 1.23	**<0.001**
SBP (mmHg)	120.73 ± 16.71	119.47 ± 16.42	129.63 ± 16.04	**<0.001**
DBP (mmHg)	72.81 ± 10.35	71.97 ± 10.08	78.77 ± 10.31	**<0.001**
ALP (U/L)	72.35 ± 23.22	71.21 ± 23.10	78.62 ± 22.92	**<0.001**
GGT (U/L)	22.00 (16.00, 31.00)	20.00 (16.00, 28.00)	32.00 (24.00, 49.00)	**<0.001**
ALT (U/L)	16.00 (12.00, 23.00)	15.00 (12.00, 21.00)	23.00 (17.00, 31.00)	**<0.001**
AST (U/L)	23.04 ± 9.53	22.67 ± 9.61	25.10 ± 8.81	**<0.001**
ALB (g/L)	44.40 ± 2.71	44.38 ± 2.70	44.53 ± 2.79	**0.035**
GLB (g/L)	29.50 ± 3.86	29.49 ± 3.81	29.58 ± 4.19	0.361
TB (μmol/L)	12.12 ± 4.95	12.08 ± 4.95	12.37 ± 4.97	**0.028**
DBIL (μmol/L)	2.29 ± 1.23	2.34 ± 1.24	1.98 ± 1.12	**<0.001**
BUN (mmol/L)	4.57 ± 1.37	4.56 ± 1.38	4.62 ± 1.29	0.066
Cr (mmol/L)	78.48 ± 25.68	77.30 ± 26.15	86.86 ± 20.16	**<0.001**
UA (μmol/L)	279.81 ± 85.92	272.40 ± 83.32	332.35 ± 85.77	**<0.001**
FBG (mg/dL)	92.58 ± 14.07	91.62 ± 12.93	99.37 ± 19.08	**<0.001**
TC (mg/dL)	178.81 ± 28.74	177.69 ± 28.30	186.76 ± 30.53	**<0.001**
TG (mg/dL)	95.58 (70.80, 133.64)	90.27 (68.14, 123.02)	161.07 (112.39, 218.60)	**<0.001**
HDL-C (mg/dL)	56.59 ± 14.07	57.62 ± 14.02	49.27 ± 12.10	**<0.001**
LDL-C (mmol/L)	87.54 ± 17.98	86.79 ± 17.91	92.82 ± 17.58	**<0.001**
SPISE	8.56 ± 1.80	8.83 ± 1.72	6.61 ± 0.94	**<0.001**
TyG	8.44 ± 0.53	8.36 ± 0.48	8.98 ± 0.54	**<0.001**
METS-IR	30.53 ± 4.61	29.77 ± 4.20	35.94 ± 3.68	**<0.001**
TG/HDL-C	1.71 (1.16, 2.69)	1.60 (1.11, 2.40)	3.33 (2.16, 5.17)	**<0.001**

Values are *n* (%) or mean ± SD or median (quartile). BMI, body mass index; DBP, diastolic blood pressure; ALP, alkaline phosphatase; SBP, systolic blood pressure; GGT, y-glutamyl transpeptidase; AST, aspartate aminotransferase; TG, triglyceride; ALB, albumin; ALT, alanine aminotransferase; GLB, globulin; LDL-C, Low-density lipid cholesterol; BUN, serum urea nitrogen; HDL-C, high-density lipoprotein cholesterol; Cr, serum creatinine; TC, total cholesterol; FBG, fasting blood glucose; UA, uric acid; DBIL, direct bilirubin; TB, total bilirubin; MASLD, metabolic dysfunction-associated steatotic liver disease; SPISE, single-point insulin sensitivity estimator; TyG, triglyceride-glucose index; METS-IR, metabolic score for insulin resistance. *p*-value less than 0.05 is expressed in bold.

Enzymatic and metabolic profiles differed by outcome. Participants who later developed MASLD had higher baseline GGT and ALT (both *p* < 0.001), along with higher TG and lower HDL-C (both *p* < 0.001). Consistent with these patterns, SPISE was reduced, whereas TyG, METS-IR, and TG/HDL-C were elevated in the incident MASLD group (all *p* < 0.001) (Table 3).

### 3.4 Longitudinal association between SPISE and risk of incident MASLD

**Figure 1:**
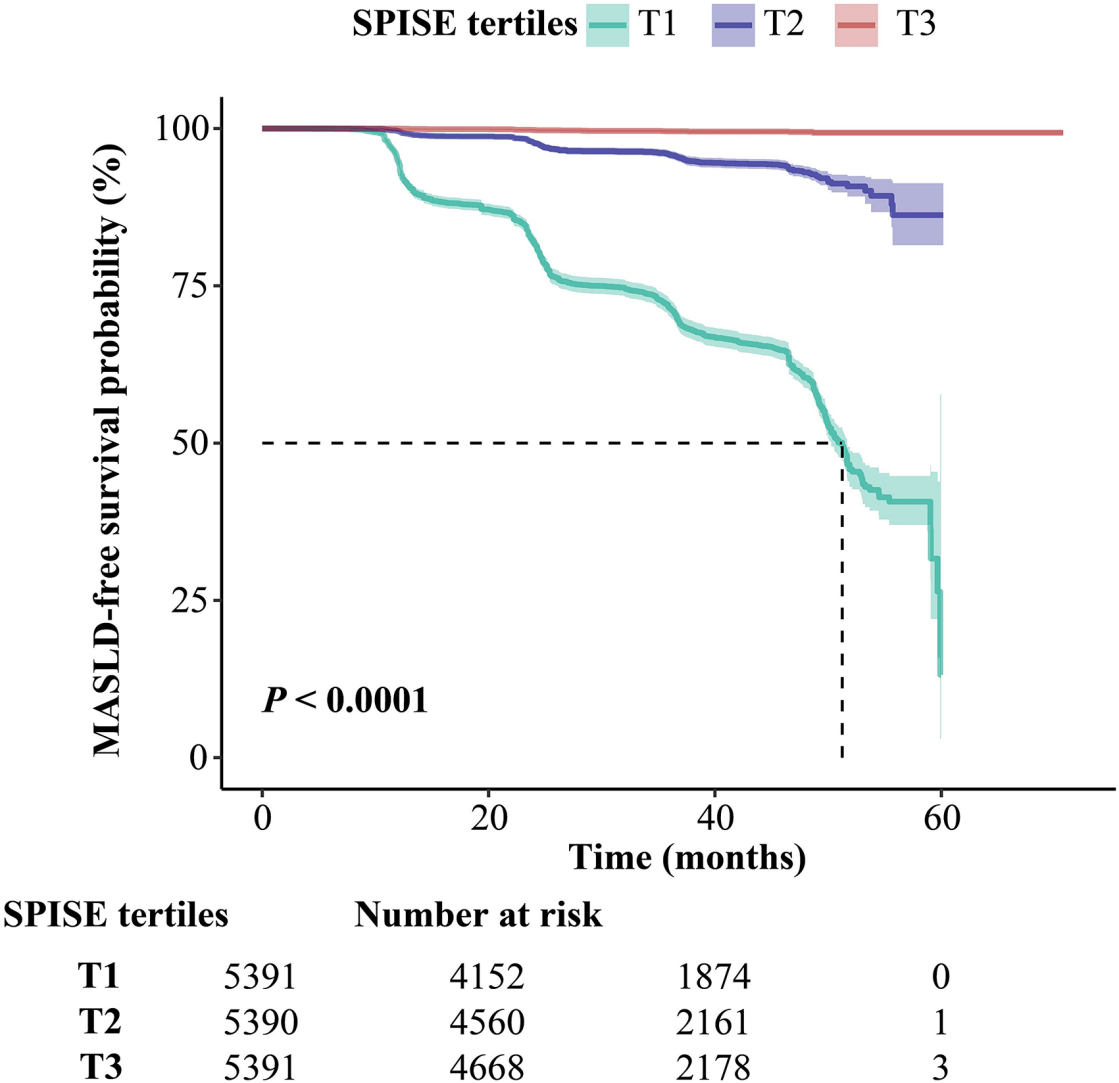
Kaplan–Meier curves for incident MASLD by SPISE tertiles. Event-free survival is shown for SPISE T1–T3. Shaded bands indicate 95% confidence intervals. *p* value was obtained from the log-rank test. Numbers at risk are provided beneath the plot. Abbreviations: MASLD, metabolic dysfunction–associated steatotic liver disease; T1–T3, tertiles.

A significant separation of Kaplan–Meier curves was observed across SPISE tertiles (log-rank *p* < 0.0001). Baseline group sizes were comparable (T1 = 5,391; T2 = 5,390; T3 = 5,391). Over time, the steepest decline in MASLD-free survival probability was observed in T1, whereas T3 maintained the highest event-free survival (Figure 1).

Cox proportional hazards analyses indicated that higher SPISE was associated with lower incident MASLD risk. For SPISE modeled continuously, the unadjusted HR was 0.39 (95% CI 0.38–0.40), and the estimate remained essentially unchanged after adjustment for age and sex (HR = 0.39, 95% CI 0.38–0.41). With additional control for liver and renal biomarkers, other laboratory indices, and blood pressure, SPISE retained an independent inverse association with incident MASLD (HR = 0.49, 95% CI 0.46–0.52) (Table 4).

**Table 4:** Longitudinal association between SPISE and the risk of MASLD.

Variable	Model 1		Model 2		Model 3	
	HR (95% CI)	*p* value	HR (95% CI)	*p* value	HR (95% CI)	*p* value
SPISE continuous	0.39 (0.38–0.40)	<0.001	0.39 (0.38–0.41)	<0.001	0.49 (0.46–0.52)	<0.001
SPISE tertiles						
T1	1.00 [Ref]		1.00 [Ref]		1.00 [Ref]	
T2	0.13 (0.12–0.15)	<0.001	0.13 (0.12–0.15)	<0.001	0.23 (0.19–0.27)	<0.001
T3	0.01 (0.01–0.02)	<0.001	0.01 (0.01–0.02)	<0.001	0.04 (0.02–0.07)	<0.001
*p* for trend	0.12 (0.11–0.14)	<0.001	0.12 (0.11–0.14)	<0.001	0.22 (0.19–0.25)	<0.001

Model 1: no covariates were adjusted. Model 2: gender, age were adjusted. Model 3: gender, age, ALP, GGT, ALT, AST, GLB, TB, ALB, DBIL, Cr, BUN, UA, SBP, DBP. BMI, body mass index; ALB, albumin; GLB, globulin; ALP, alkaline phosphatase; GGT, gamma-glutamyl transferase; AST, aspartate aminotransferase; ALT, alanine aminotransferase; LDL-C, low-density lipoprotein cholesterol; HDL-C, high-density lipoprotein cholesterol; DBIL, direct bilirubin; Cr, creatinine; BUN, blood urea nitrogen; UA, uric acid; TB, total bilirubin; SBP, systolic blood pressure; DBP, diastolic blood pressure; MASLD, metabolic dysfunction-associated steatotic liver disease; SPISE, single-point insulin sensitivity estimator.

Tertile analyses showed a clear gradient. Using T1 as the reference, the fully adjusted HRs were 0.23 (95% CI 0.19–0.27) for T2 and 0.04 (95% CI 0.02–0.07), with a significant trend across categories (*p* for trend < 0.001) (Table 4).

### 3.5 Nonlinear exposure–response pattern for SPISE and MASLD risk

**Figure 2:**
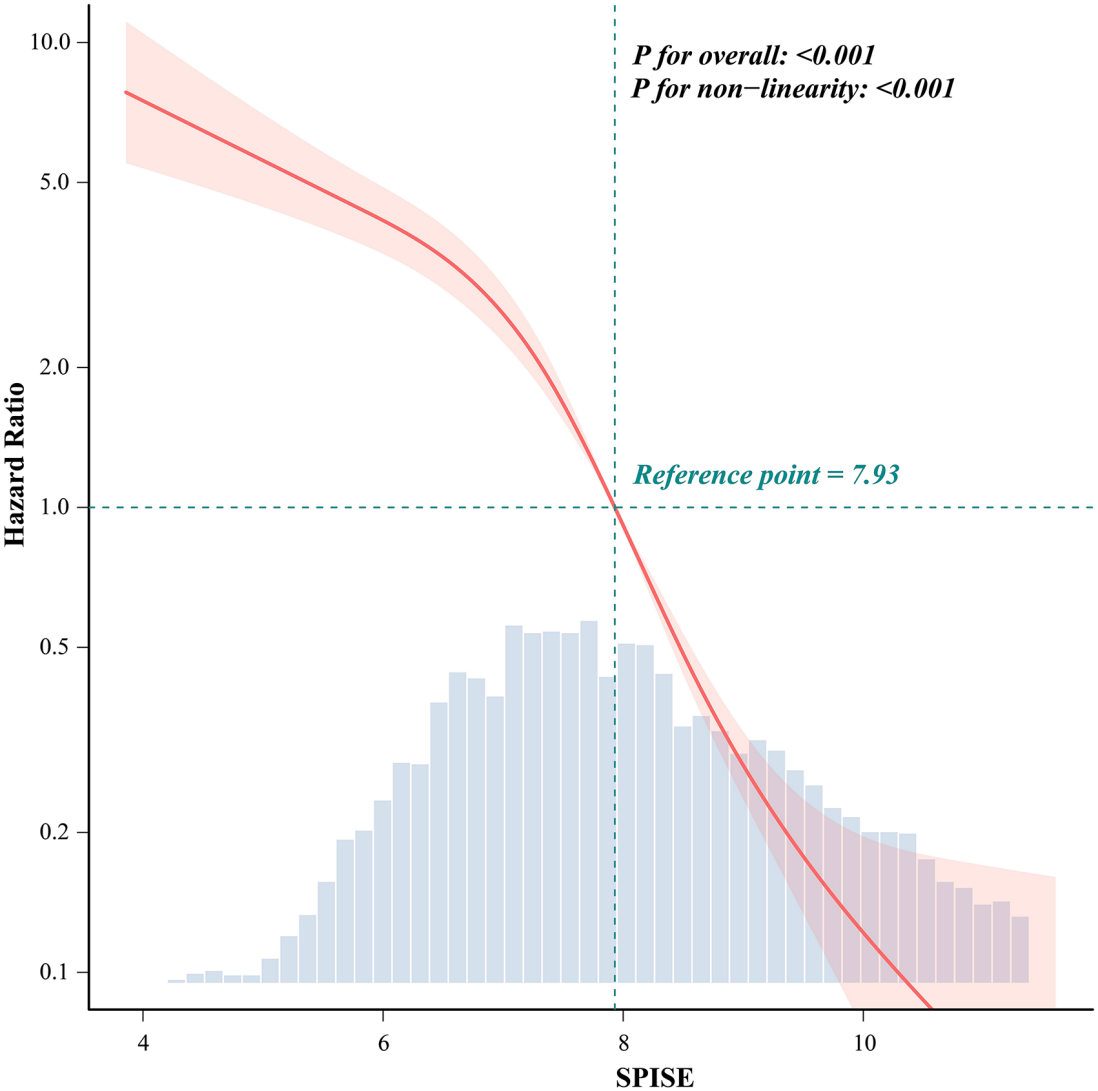
Restricted cubic spline association between SPISE and incident MASLD risk. Hazard ratios across the SPISE range were estimated using restricted cubic splines in the fully adjusted Cox model. The solid line represents the adjusted hazard ratio and the shaded band indicates the 95% confidence interval. SPISE = 7.93 was used as the reference (HR = 1.0). The histogram shows the distribution of SPISE. MASLD, metabolic dysfunction–associated steatotic liver disease; HR, hazard ratio; SPISE, single-point insulin sensitivity estimator.

Restricted cubic spline analyses indicated a significant nonlinear association between SPISE and incident MASLD risk (overall *p* < 0.001; *p* for non-linear < 0.001). Using SPISE = 7.93 as the reference (HR = 1.0), risk increased more steeply at SPISE values below the reference point, whereas risk continued to decrease above the reference range with a progressively attenuated slope (Figure 2).

### 3.6 Incremental predictive value compared with TyG, METS-IR, and TG/HDL-C

When added individually to the fully adjusted base model, SPISE yielded the largest improvements in risk reclassification and discrimination (NRI = 0.363, 95% CI 0.314–0.399; IDI = 0.093, 95% CI 0.077–0.109; both *p* < 0.001). By comparison, adding TyG resulted in NRI = 0.179 and IDI = 0.032 (both *p* < 0.001); adding METS-IR resulted in NRI = 0.214 and IDI = 0.089 (both *p* < 0.001). Adding TG/HDL-C did not significantly improve reclassification (NRI = 0.025, *p* = 0.391), although a small improvement in discrimination was observed (IDI = 0.010, *p* = 0.016) (Table 5).

**Table 5:** Incremental predictive performance of SPISE, TyG, METS-IR, and TG/HDL-C for incident MASLD: NRI and IDI analyses.

Event	NRI (95% CI)	NRI *p* value	IDI (95% CI)	IDI *p* value
Baseline model	Ref		Ref	
Baseline model + SPISE	0.363 (0.314 to 0.399)	<0.001	0.093 (0.077 to 0.109)	<0.001
Baseline model + TyG	0.179 (0.117 to 0.219)	<0.001	0.032 (0.020 to 0.044)	<0.001
Baseline model + METS-IR	0.214 (0.152 to 0.268)	<0.001	0.089 (0.073 to 0.106)	<0.001
Baseline model + TG/HDL	0.025 (−0.054 to 0.096)	0.391	0.010 (0.003 to 0.017)	0.016

SPISE, single-point insulin sensitivity estimator; TyG, triglyceride-glucose index; METS-IR, metabolic score for insulin resistance; TG, triglyceride; HDL, high-density lipoprotein cholesterol; NRI, net reclassification improvement; IDI, integrated discrimination improvement.

### 3.7 Variable selection and multivariable modeling: identification of key predictors

**Figure 3:**
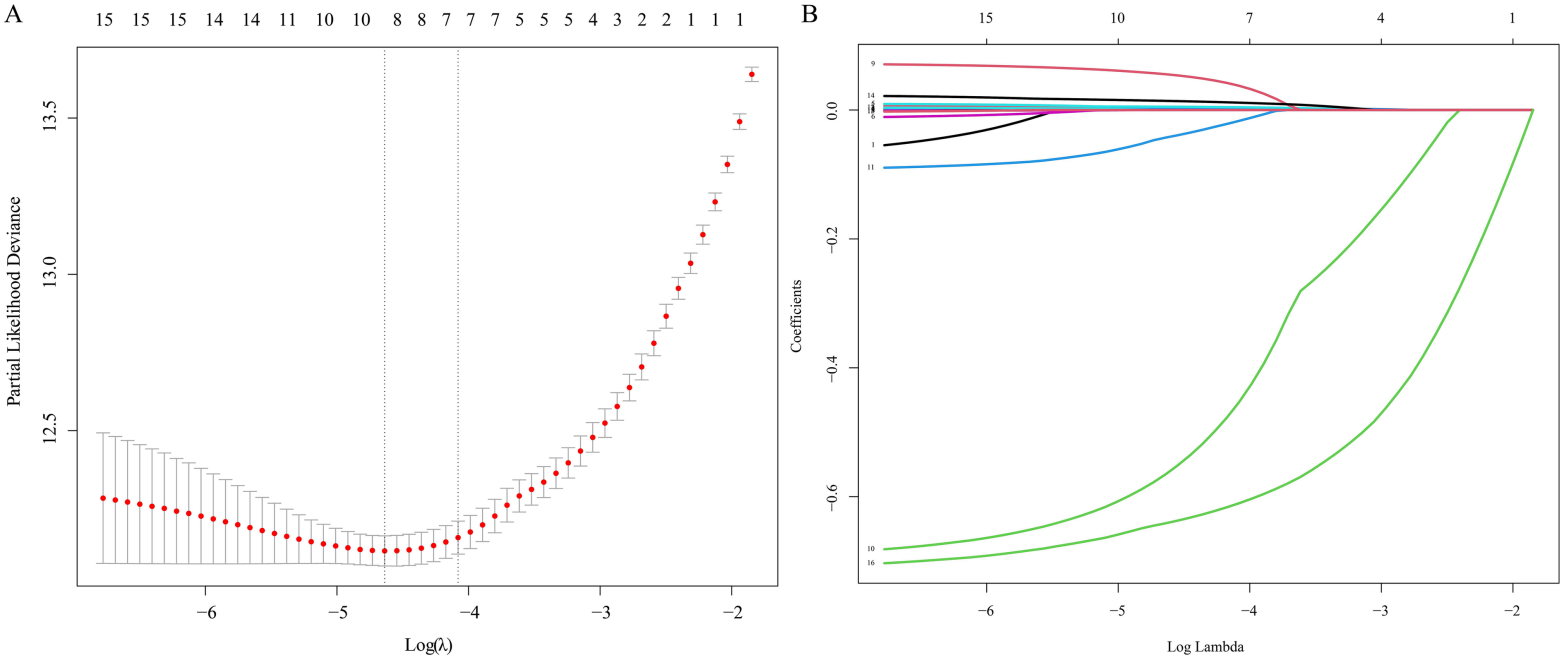
Predictor selection using LASSO regression for incident MASLD risk modeling. **(A)** Cross-validated partial likelihood deviance plotted against log(*λ*); vertical dashed lines indicate the optimal *λ* values. **(B)** Coefficient trajectories of candidate predictors as a function of log(*λ*). MASLD, metabolic dysfunction–associated steatotic liver disease; LASSO, least absolute shrinkage and selection operator; SPISE, single-point insulin sensitivity estimator; *λ*, penalty parameter.

Using LASSO applied to candidate variables from the fully adjusted model together with SPISE, seven variables with non-zero coefficients were identified at the cross-validated optimal *λ* (Figure 3). Subsequent Cox regression analyses retained six independent predictors in the multivariable model (Table 6): SPISE, GGT, ALT, total bilirubin (TB), direct bilirubin (Dbil), and DBP. SPISE was inversely associated with incident MASLD (HR = 0.50, 95% CI 0.47–0.53). ALT and DBP were positively associated with risk, whereas Dbil was inversely associated. Although BUN was associated with the outcome in univariable analysis, the association did not remain independent in multivariable models and was not included in the final prediction model (Table 6).

**Table 6:** Univariable and multivariable cox regression analyses for incident MASLD.

Variables	Univariate analysis		Multivariate analysis	
	HR (95% CI)	*p* value	HR (95% CI)	*p* value
GGT	1.01 (1.01–1.01)	<0.001	1.00 (1.00–1.00)	0.017
ALT	1.01 (1.01–1.01)	<0.001	1.01 (1.01–1.01)	<0.001
TB	1.00 (0.99–1.02)	0.620	1.08 (1.07–1.08)	<0.001
DBIL	0.53 (0.49–0.57)	<0.001	0.47 (0.43–0.51)	<0.001
BUN	0.93 (0.90–0.97)	<0.001		
DBP	1.06 (1.05–1.06)	<0.001	1.02 (1.01–1.03)	<0.001
SPISE	0.39 (0.37–0.41)	<0.001	0.50 (0.47–0.53)	<0.001

GGT, y-glutamyl transpeptidase; ALT, alanine aminotransferase; DBIL, direct bilirubin; TB, total bilirubin; BUN, blood urea nitrogen; DBP, Diastolic blood pressure; SPISE, single-point insulin sensitivity estimator; HR, hazard Ratio; CI, confidence Interval. *p*-value less than 0.05 is expressed in bold.

### 3.8 Predictive performance and clinical utility

A nomogram was constructed using the six predictors in the final multivariable Cox model (SPISE, GGT, ALT, TB, Dbil, and DBP) (Supplementary Figure S1A), and an online dynamic prediction tool was developed to estimate individualized 12- and 24-month risks of incident MASLD (Supplementary Figure S2). Time-dependent ROC analysis demonstrated good discrimination for mid-term MASLD risk (AUC 0.859 at 12 months and 0.886 at 24 months) (Supplementary Figure S1B). At 24 months, decision curve analysis showed that the model achieved greater net benefit than both “treat-all” and “treat-none” over an approximate threshold probability range of 0.06–0.82. For threshold probabilities <0.06, the “treat-all” strategy yielded higher net benefit; for threshold probabilities >0.82, model net benefit approached zero and was not superior to the baseline strategies (Supplementary Figure S1C).

### 3.9 Sensitivity analysis

As a sensitivity analysis, participants who developed MASLD within the first 12 months of follow-up were excluded and the main analyses were repeated. The inverse association between SPISE and incident MASLD remained materially unchanged. In the fully adjusted Cox model, continuous SPISE remained significantly associated with a lower risk of incident MASLD (HR = 0.51, 95% CI 0.47–0.54, *p* < 0.001). In tertile analyses, compared with T1, the fully adjusted HRs were 0.26 (95% CI 0.21–0.32) for T2 and 0.05 (95% CI 0.03–0.08) for T3, with *p* for trend < 0.001 (Supplementary Table S1). Restricted cubic spline analysis also showed a persistent nonlinear association between SPISE and incident MASLD risk (*p* for overall < 0.001; *p* for non-linearity < 0.001), supporting the robustness of the primary findings (Supplementary Figure S3).

### 3.10 Internal validation of the prediction model

To further evaluate model robustness, internal cross-validation was performed for the 12- and 24-month prediction models. For the 12-month model, the mean cross-validated AUC was 0.852 and the mean Brier score was 0.038 (Supplementary Table S2 and Figure S4). For the 24-month model, the mean cross-validated AUC was 0.876 and the mean Brier score was 0.094 (Supplementary Table S2 and Figure S5). These values were close to the apparent model performance estimates, indicating stable discrimination and predictive accuracy with minimal evidence of overfitting.

## 4 Discussion

A two-stage framework (cross-sectional discovery plus longitudinal validation) was applied to systematically evaluate the associations of SPISE with MASLD prevalence and incident risk, to quantify its incremental predictive value, and to assess the translational potential of SPISE-based risk modeling. Findings were directionally consistent across study stages. After comprehensive confounder adjustment, SPISE was stably and inversely associated with prevalent MASLD. In the follow-up cohort, higher baseline SPISE independently predicted a lower risk of incident MASLD, supporting SPISE as a metabolic phenotype for mid-term risk identification. Compared with TyG, METS-IR, and TG/HDL-C, inclusion of SPISE yielded larger improvements in risk reclassification and discrimination within the same baseline modeling framework, indicating more prominent incremental predictive utility. Furthermore, key predictors identified by LASSO and Cox regression (SPISE, GGT, ALT, TB, Dbil, and DBP) were integrated into an online prediction model. The model demonstrated strong discrimination at both 12 and 24 months and yielded favorable net benefit across clinically meaningful risk thresholds, supporting its potential use for early MASLD risk stratification and tiered management.

These findings are consistent with prior work identifying insulin resistance as a key driver of MASLD onset and progression (1, 2). Insulin resistance can promote MASLD through enhanced lipolysis, increased hepatic influx of free fatty acids, and imbalance between hepatic lipogenesis and lipid oxidation, leading to hepatic lipid accumulation and disease progression. SPISE, derived from routine measures (BMI, TG, and HDL-C), can approximate an insulin-sensitivity phenotype at the population level. The observed inverse association between SPISE and MASLD risk suggests that a composite phenotype characterized by impaired insulin sensitivity, dyslipidemia, and increased adiposity burden may be particularly relevant to early MASLD risk formation (29). Prior studies have reported that higher MASH activity in T2DM is associated with more pronounced insulin resistance and adipose tissue dysfunction (30), and the Swedish SCAPIS study has linked obesity, T2DM, hypertension, and other insulin resistance–related abnormalities to MASLD prevalence and severity (2). In this context, the longitudinal findings provide temporal support for these associations by linking baseline insulin-sensitivity phenotypes to subsequent MASLD risk. In addition, a US study reported a positive association between the monocyte-to-lymphocyte ratio (MLR) and MASLD risk (31), consistent with the concept that metabolic inflammation contributes to insulin resistance and hepatic steatosis and complements the mechanistic understanding of MASLD from a systemic metabolic dysregulation perspective.

From a mechanistic standpoint, the inverse association between SPISE and MASLD risk is plausibly driven by multiple coupled pathways. Insulin resistance can increase adipose lipolysis and augment the flux of free fatty acids to the liver, resulting in hepatic lipid overload. Excess lipids not only enhance triglyceride synthesis but may also induce hepatocellular injury and inflammation via lipotoxicity, oxidative stress, and inflammasome activation, thereby promoting progression from simple steatosis to MASH (32). Metabolomics evidence indicates that progression to MASH is accompanied by increases in inflammation-related metabolites and downregulation of fatty acid degradation and amino-acid metabolic pathways (32), consistent with downstream consequences of dysregulated insulin signaling. Insulin resistance is also frequently accompanied by adipokine imbalance and chronic low-grade inflammation (e.g., reduced adiponectin and elevated pro-inflammatory cytokines), which together create a microenvironment favoring lipid deposition and fibrosis (33). Multi-organ crosstalk, including the gut–liver axis, may further amplify inflammatory and metabolic perturbations (34–36). Moreover, impaired mitochondrial biogenesis and function may reduce *β*-oxidation capacity and increase reactive oxygen species generation, aggravating oxidative stress and hepatocellular injury (37). Collectively, these mechanisms support the biological plausibility of a stable association between the insulin-sensitivity phenotype reflected by SPISE and MASLD risk.

A notable finding was the superior improvement in discrimination and reclassification associated with SPISE compared with TyG, METS-IR, and TG/HDL-C. This advantage may reflect closer alignment with key MASLD-relevant metabolic phenotypes. SPISE integrates adiposity burden (BMI) and characteristic insulin resistance–related lipid features (high TG and low HDL-C), consistent with a composite pathway linking adiposity, dyslipidemia, and impaired insulin sensitivity. In contrast, TyG may more strongly emphasize glyco-lipid status, TG/HDL-C provides a narrower information dimension, and METS-IR integrates multiple metabolic variables with different compositions and weights, potentially leading to performance variability across populations and outcome definitions (26). Prospective evidence from the UK Biobank also suggests heterogeneity in association strength and discriminatory capacity across different insulin resistance indices for liver-related outcomes, with potential subgroup differences (26). Therefore, the relative advantage observed for SPISE may indicate greater sensitivity for capturing adiposity- and dyslipidemia-dominant insulin resistance phenotypes, but external validation across populations and MASLD definitions remains necessary.

These findings have potential clinical and translational implications. SPISE can be calculated from routine anthropometric and lipid measures with low cost and high feasibility, supporting use in health examinations and primary care settings as an initial risk-screening phenotype for high-risk groups (e.g., metabolic syndrome and T2DM). In addition, the developed multivariable online model integrates SPISE with readily available liver biochemistry and blood pressure measures to provide individualized mid-term risk estimates. Such estimates may facilitate stratified management pathways, in which higher-risk individuals are prioritized for liver-specific assessment (e.g., vibration-controlled transient elastography) to further evaluate steatosis and fibrosis risk (38, 39). This approach may improve allocation efficiency in resource-limited settings by reserving higher-cost or specialized testing for those most likely to benefit. Prior work in regions such as South Asia has attempted to optimize referral pathways using FIB-4 and diabetes-related clinical information to improve the cost-effectiveness of elastography (39); the present model may serve as a complementary stratification tool within similar workflows, although clinical benefit requires confirmation through external validation and implementation studies. As a repeatable metabolic phenotype, SPISE may also be useful for monitoring metabolic improvement following lifestyle or pharmacologic interventions and for exploring corresponding changes in MASLD risk (40).

Several limitations should be acknowledged. First, despite strengthened temporality from the longitudinal design, residual confounding cannot be excluded in observational analyses; causal inference is therefore not supported. Subclinical steatosis may also exert early reverse effects on insulin-sensitivity phenotypes. Second, MASLD was defined on the basis of ultrasound-confirmed steatosis. Although abdominal ultrasonography is practical and widely used in population-based studies, its sensitivity is limited for detecting mild steatosis and its diagnostic performance is operator-dependent. Therefore, some degree of outcome misclassification may have occurred. Such non-differential misclassification could have attenuated the observed associations and may also have influenced the apparent predictive performance and generalizability of the model. Accordingly, the present model should be interpreted as predicting ultrasound-defined MASLD, and validation in cohorts using more sensitive imaging modalities or histological standards is warranted. Third, the ethnic and geographic composition of the study populations was relatively homogeneous; given inter-population differences in MASLD prevalence and phenotypes (31, 41), generalizability of the associations and model performance requires external validation across regions, ethnicities, and clinical settings, with systematic evaluation of calibration and potential performance drift. Fourth, some biochemical predictors (e.g., ALT and GGT) have limited specificity and may be influenced by alcohol intake, medications, and comorbidities. Future work could explore incorporation of more liver pathology–specific biomarkers (e.g., cytokeratin-18 and procollagen III peptide) or emerging protein markers (e.g., IGFBP7) to enhance interpretability and precision (42). Finally, model evaluation primarily focused on discrimination and decision curve analysis; further external validation, calibration assessment, and prospective implementation studies are needed to determine net benefit within real-world referral and management pathways and to clarify effects on long-term outcomes.

## 5 Conclusion

Across both cross-sectional and longitudinal analyses, higher SPISE was independently associated with lower odds of prevalent MASLD and a lower risk of incident MASLD, supporting SPISE as a simple and low-cost metabolic phenotype for MASLD risk identification. A SPISE-based online prediction model incorporating liver biochemistry and blood pressure demonstrated good mid-term predictive performance and may support early screening and stratified management. Multicenter external validation and prospective implementation studies are required before broader clinical adoption.

## Data Availability

The original contributions presented in the study are included in the article/Supplementary material, further inquiries can be directed to the corresponding author.
