# Supplementary material for: Association of SPISE with prevalent and incident MASLD: a two-stage population-based study and development of a risk prediction model
Source: Front Nutr. 2026 Apr 29;13:1811730. doi: 10.3389/fnut.2026.1811730 (PMC13167443; doi:10.3389/fnut.2026.1811730)
Supplement: Supplementary file 1 [file Table_1.docx]

Supplementary Material

**Table S1** Sensitivity analysis of the longitudinal association between SPISE and incident MASLD after exclusion of participants who developed MASLD within the first 12 months of follow-up.

**Table S2** Internal validation performance of the 12- and 24-month Cox prediction models.

**Figure S1** Development and performance of the SPISE-based prediction model for incident MASLD.

**Figure S2** Screenshot of the web-based dynamic nomogram for individualized prediction of incident MASLD.

**Figure S3** Restricted cubic spline analysis of the association between SPISE and incident MASLD after exclusion of participants who developed MASLD within the first 12 months of follow-up.

**Figure S4** Cross-validation results for the 12-month Cox prediction model: distributions of AUC and Brier score.

**Figure S5** Cross-validation results for the 24-month Cox prediction model: distributions of AUC and Brier score.

| **Table S1.** Sensitivity analysis of the longitudinal association between SPISE and incident MASLD after exclusion of participants who developed MASLD within the first 12 months of follow-up | | | | | | | | |
| --- | --- | --- | --- | --- | --- | --- | --- | --- |
|  |  |  |  |  |  |  |  |  |
| **Variable** | **Model 1** | |  | **Model 2** | |  | **Model 3** | |
|  | **HR (95% CI)** | ***P*** value |  | **HR (95% CI)** | ***P*** value |  | **HR (95% CI)** | ***P*** value |
| **SPISE continuous** | 0.39 (0.38 - 0.41) | <0.001 |  | 0.39 (0.38 - 0.41) | <0.001 |  | 0.51 (0.47 - 0.54) | <0.001 |
| **SPISE tertiles** |  |  |  |  |  |  |  |  |
| T1 | 1.00 [Ref] | |  | 1.00 [Ref] | |  | 1.00 [Ref] | |
| T2 | 0.14 (0.12 - 0.16) | <0.001 |  | 0.14 (0.12 - 0.16) | <0.001 |  | 0.26 (0.21 - 0.32) | <0.001 |
| T3 | 0.01 (0.01 - 0.02) | <0.001 |  | 0.01 (0.01 - 0.02) | <0.001 |  | 0.05 (0.03 - 0.08) | <0.001 |
| *P* for trend | 0.13 (0.12 - 0.15) | <0.001 |  | 0.13 (0.12 - 0.15) | <0.001 |  | 0.24 (0.21 - 0.29) | <0.001 |
| Model 1: no covariates were adjusted Model 2: Gender, Age were adjusted Model 3: Gender, Age, ALP, GGT, ALT, AST, GLB, TB, ALB, DBIL, Cr, BUN, UA, SBP,DBP. Abbreviations: BMI, body mass index; ALB, albumin; GLB, globulin; ALP, alkaline phosphatase; GGT, gamma-glutamyl transferase; AST, aspartate aminotransferase; ALT, alanine aminotransferase; LDL-C, low-density lipoprotein cholesterol; HDL-C, high-density lipoprotein cholesterol; DBIL, direct bilirubin; Cr, creatinine; BUN, blood urea nitrogen; UA, uric acid; TB, total bilirubin; SBP, systolic blood pressure; DBP, diastolic blood pressure; MASLD, metabolic dysfunction-associated steatotic liver disease; SPISE, single-point insulin sensitivity estimator. | | | | | | | | |
|  |  |  |  |  |  |  |  |  |
|  |  |  |  |  |  |  |  |  |
|  |  |  |  |  |  |  |  |  |
|  |  |  |  |  |  |  |  |  |
|  |  |  |  |  |  |  |  |  |
|  |  |  |  |  |  |  |  |  |
|  |  |  |  |  |  |  |  |  |
|  |  |  |  |  |  |  |  |  |
|  |  |  |  |  |  |  |  |  |

| **Table S2.** Internal validation performance of the 12- and 24-month Cox prediction models | | |
| --- | --- | --- |
|  |  |  |
| **Prediction horizon** | **AUC** | **Brier score** |
| 12-month | 0.852 | 0.038 |
| 24-month | 0.876 | 0.094 |
| Notes: Values are mean cross-validated estimates. Higher AUC indicates better discrimination, whereas lower Brier score indicates better overall predictive accuracy. Internal validation was performed using repeated 5-fold cross-validation. | | |
|  |  |  |
|  |  |  |
|  |  |  |
|  |  |  |


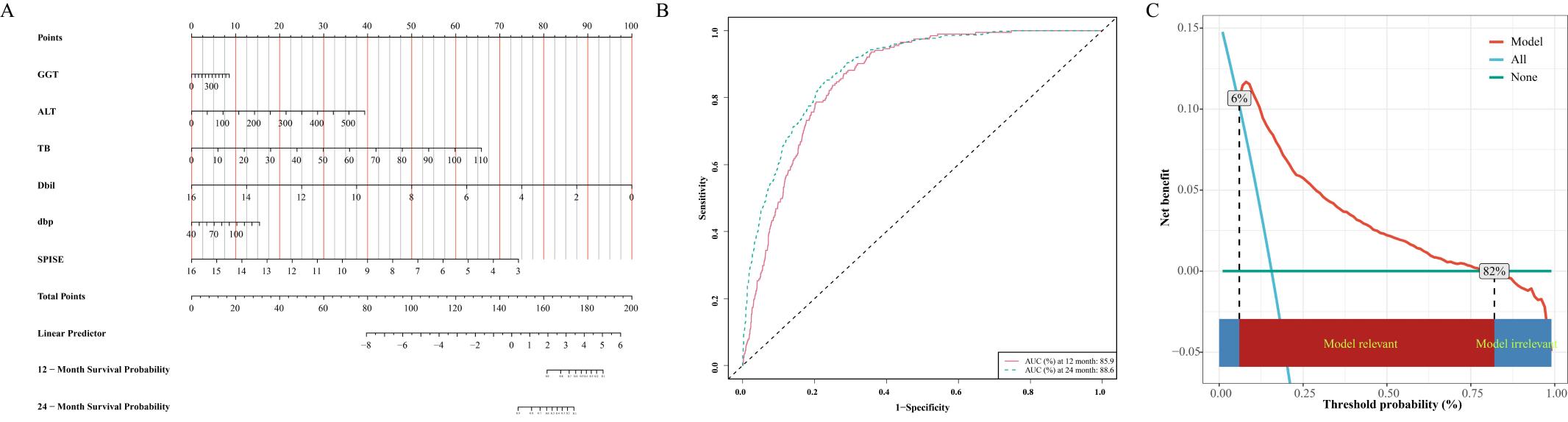


**Figure S1. Development and performance of the SPISE-based prediction model for incident MASLD.** (A) Nomogram constructed from the final multivariable Cox model. (B) Time-dependent ROC curves at 12 and 24 months with corresponding AUC values. (C) Decision curve analysis at 24 months comparing net benefit of the model with “treat-all” and “treat-none” strategies across threshold probabilities. Abbreviations: AUC, area under the curve; DCA, decision curve analysis; MASLD, metabolic dysfunction–associated steatotic liver disease; ROC, receiver operating characteristic; SPISE, single-point insulin sensitivity estimato.


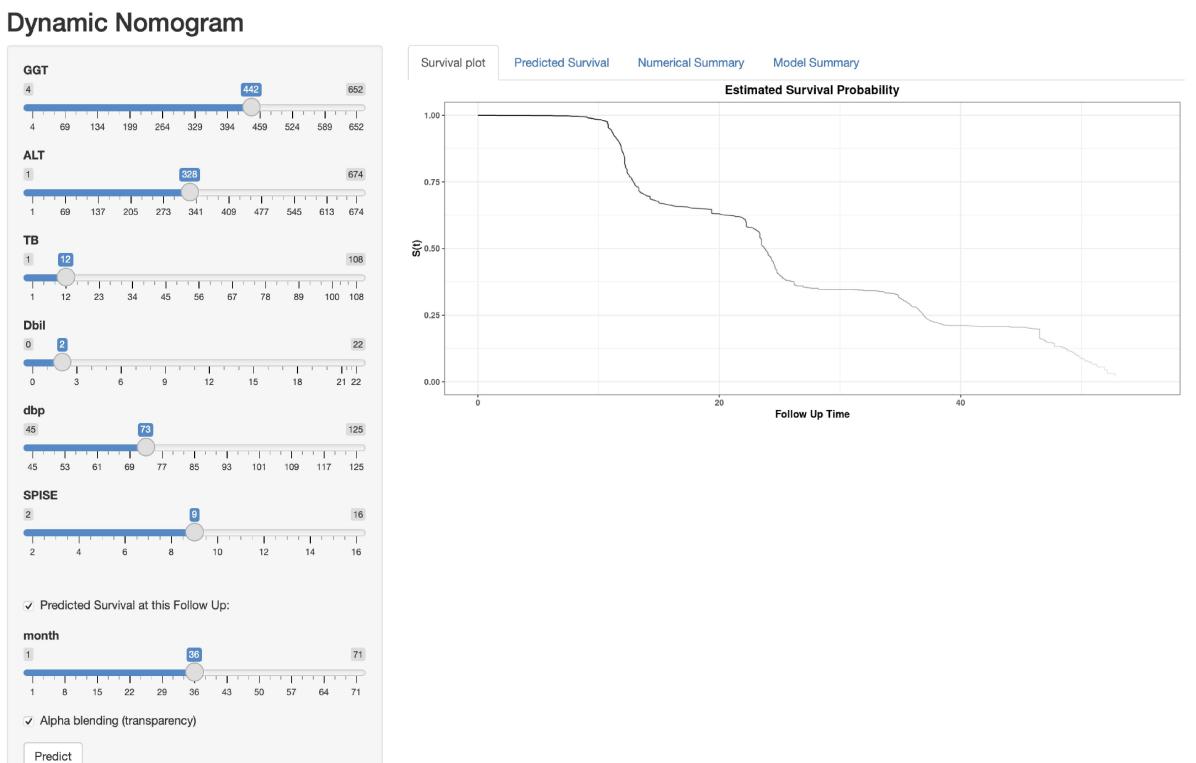


**Figure S2** Screenshot of the web-based dynamic nomogram for individualized prediction of incident MASLD.


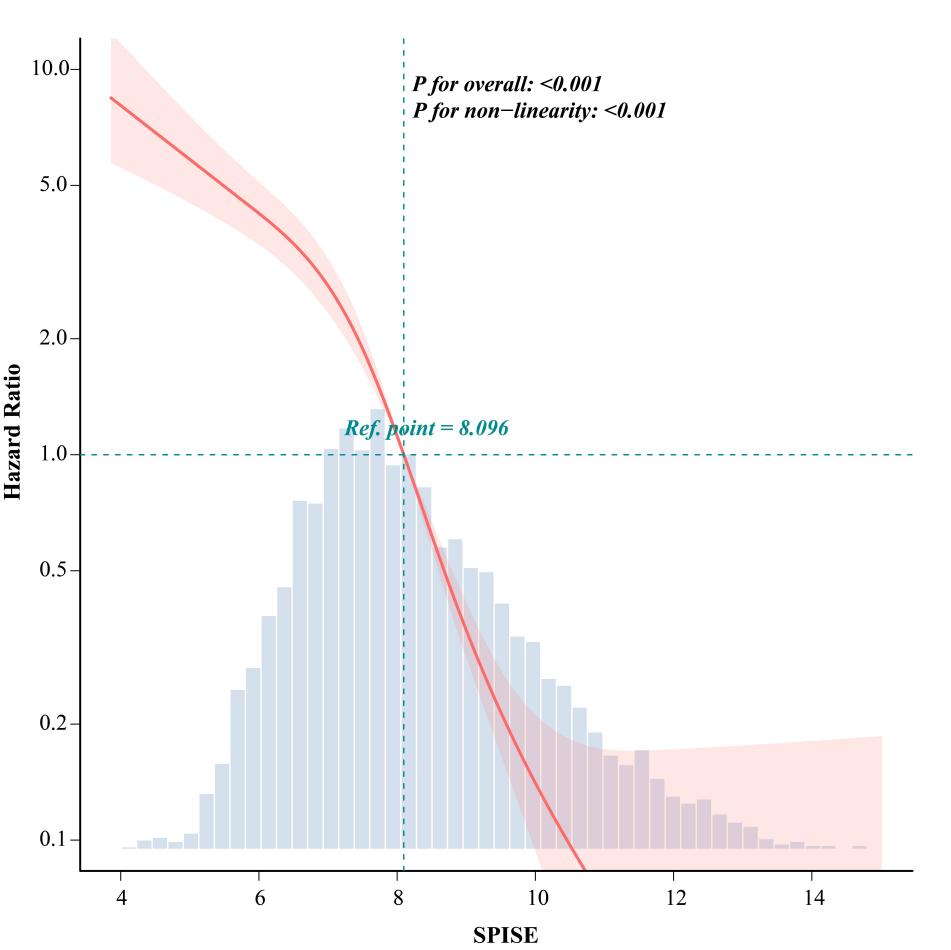


**Figure S3** Restricted cubic spline analysis of the association between SPISE and incident MASLD after exclusion of participants who developed MASLD within the first 12 months of follow-up.


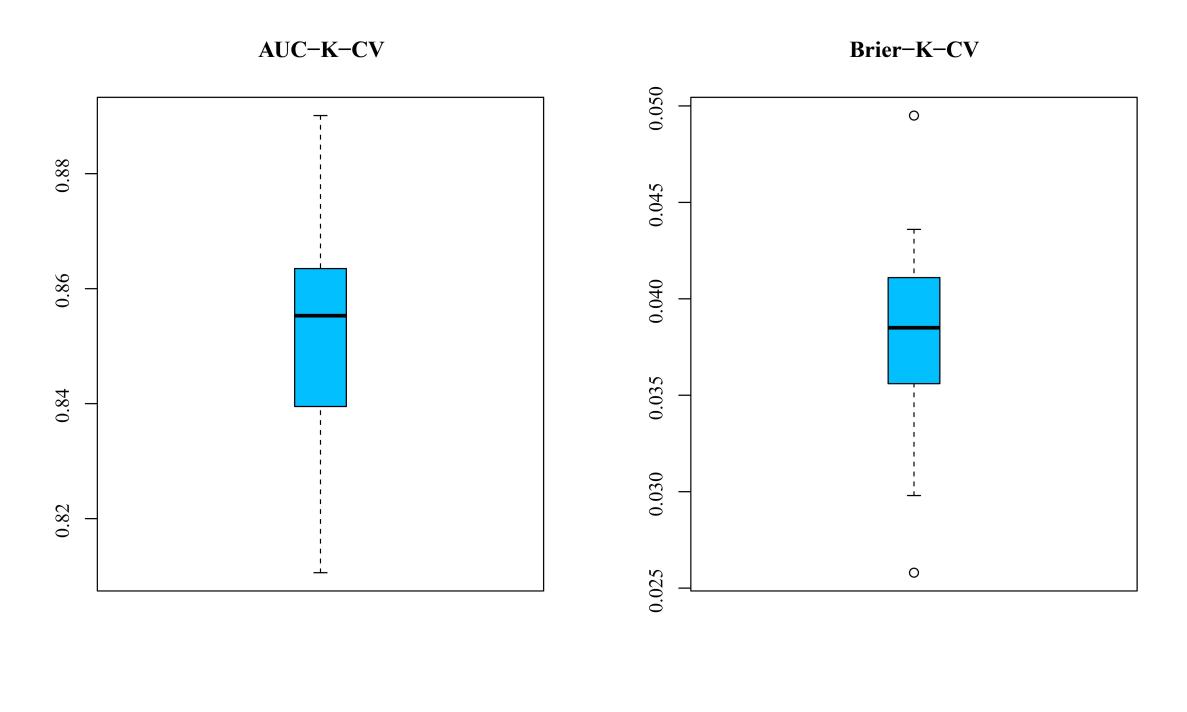


**Figure S4** Cross-validation results for the 12-month Cox prediction model: distributions of AUC and Brier score.


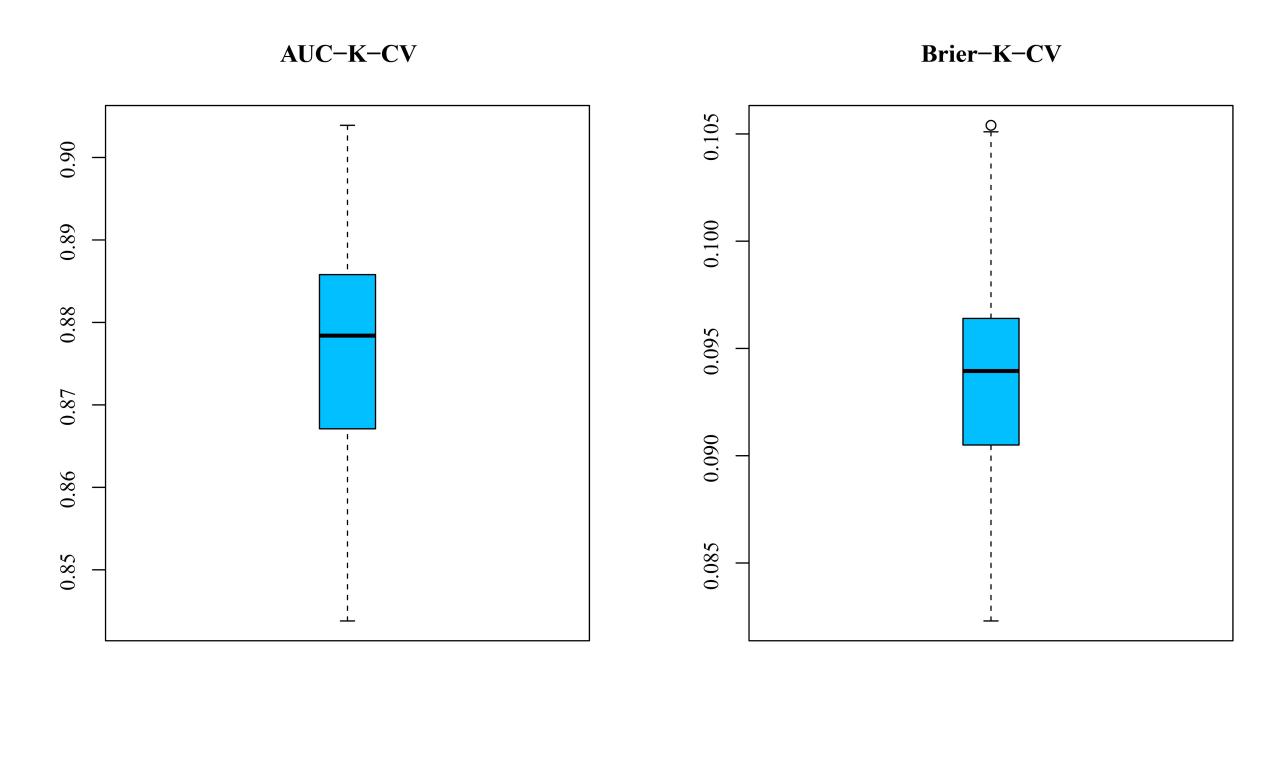


**Figure S5** Cross-validation results for the 24-month Cox prediction model: distributions of AUC and Brier score.
